# A Novel Al–Cu Composite with Ultra‐High Strength at 350 °C via Dual‐Phase Particle Reinforced Submicron‐Structure

**DOI:** 10.1002/advs.202207208

**Published:** 2023-07-11

**Authors:** Kewei Xie, Jinfeng Nie, Chang Liu, Wenhao Cha, Ge Wu, Xiangfa Liu, Sida Liu

**Affiliations:** ^1^ Key Laboratory for Liquid‐Solid Structural Evolution and Processing of Materials Ministry of Education Shandong University Jinan 250061 China; ^2^ Nano and Heterogeneous Materials Center School of Materials Science and Engineering Nanjing University of Science and Technology Nanjing 210094 China; ^3^ Center for Alloy Innovation and Design (CAID) State Key Laboratory for Mechanical Behavior of Materials Xi'an Jiaotong University Xi'an 710049 China; ^4^ Faculty of Georesources and Materials Engineering RWTH Aachen University 52056 Aachen Germany; ^5^ Center for Advancing Materials Performance from the Nanoscale and Hysitron Applied Research Center in China State Key Laboratory for Mechanical Behavior of Materials Xi'an Jiaotong University Xi'an 710049 China; ^6^ Laboratory for multiscale mechanics and medical science SV LAB School of Aerospace Xi'an Jiaotong University Xi'an 710049 China

**Keywords:** aluminum matrix composites, Guinier–Preston zones, high‐temperature strength, nano‐AlN particles, submicron‐Al_2_O_3_ particles

## Abstract

Thermal stability determines a material's ability to maintain its performance at desired service temperatures. This is especially important for aluminum (Al) alloys, which are widely used in the commercial sector. Herein, an ultra‐strong and heat‐resistant Al‐Cu composite is fabricated with a structure of nano‐AlN and submicron‐Al_2_O_3_ particles uniformly distributed in the matrix. At 350 °C, the (8.2AlN+1Al_2_O_3_)_p_/Al‐0.9Cu composite achieves a high strength of 187 MPa along with a 4.6% ductility under tension. The high strength and good ductility benefit from strong pinning effect on dislocation motion and grain boundary sliding by uniform dispersion of nano‐AlN particles, in conjunction with the precipitation of Guinier–Preston (GP) zones, enhancing strain hardening capacity during plastic deformation. This work can expand the selection of Al–Cu composites for potential applications at service temperatures as high as ≈350 °C.

## Graphical abstract

An ultra‐strong and heat‐resistant Al‐Cu composite is fabricated, showing a structure of nano‐AlN and submicron‐Al_2_O_3_ particles uniformly distributed in the matrix. The deformation mechanism at elevated temperatures is studied, which reveals that grain boundary sliding is suppressed and dislocation multiplication is favored due to the pinning effect by nano‐AlN particles and Guinier–Preston zones.

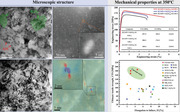

## 1 Introduction

Aluminum (Al) alloys have wide room‐temperature applications in transportation and aerospace industries due to their low weights, high specific strengths, and outstanding corrosion resistance.^[^ ^1^, ^2^, ^3^, ^4^, ^5^ ^]^ Improving the energy efficiency and reducing greenhouse gas emissions have been among the central topics related to the environment and climate change in recent years, and this creates higher requirements for the heat resistance and thermal stability of alloys. For traditional precipitation‐hardened Al alloys, however, a major problem is that the microstructures are unstable and the precipitated phases are highly prone to coarsening at temperatures above 200 °C.^[^ ^6^, ^7^, ^8^, ^9^, ^10^ ^]^ This results in a substantial loss of strength, and the alloys cannot meet the service requirements. Therefore, the development of Al matrix composites with high heat resistance and high specific strengths is urgently needed. Researchers have carried out several studies to improve the high‐temperature mechanical properties of Al alloys. These include alloying Al with various elements, especially transition and rare‐earth metals, to generate thermally stable microstructures in the matrix, as well as exploring parallel efforts by using novel alloy processing methods, such as powder metallurgy, additive manufacturing and rapid solidification, post‐casting treatments, and engineering alloys in a liquid state prior to casting.^[^ ^11^, ^12^, ^13^, ^14^, ^15^, ^16^, ^17^, ^18^ ^]^

Herein, we report a “fine grain size with a uniform dispersion of thermally stable, small, hard particles within the matrix and on the grain boundaries” method to achieve excellent heat‐resistant mechanical properties in Al matrix composites with different Cu contents. According to our previous study,^[^ ^19^ ^]^ the addition of submicron‐Al_2_O_3_ particles can play an important role in making the agglomerated nano‐AlN particles distributed more uniformly in the Al matrix, and then the strengthening effect played by the nano‐AlN particles can be effectively guaranteed. In the present work, utilizing a liquid–solid reaction method and subsequent hot extrusion process, we achieved a homogeneous distribution of high‐density nano‐AlN particles and submicron‐Al_2_O_3_ particles. The uniformly dispersed intragranular nano‐AlN particles promote a strengthening effect through dislocation–nanoparticle interactions, besides, the nano‐AlN particles located at the grain boundaries (GBs) restrict the GBs from sliding and coarsening during plastic deformation. In addition, a large quantity of nano‐precipitated GP zones interacted with dislocations, thereby providing an exceptional tensile strength at elevated temperatures. We demonstrate the “fine grain size with a uniform dispersion of thermally stable, small, hard particles within the matrix and on the grain boundaries” strategy by introducing nano‐AlN particles and submicron‐Al_2_O_3_ particles into a submicron‐grain Al matrix with different Cu contents. Combined with the appropriate T6 heat treatment, a large number of dispersed nanoclusters, that is, GP zones, were precipitated in the (8.2AlN+1Al_2_O_3_)_p_/Al‐0.9Cu composite matrix. As a result, the (8.2AlN+1Al_2_O_3_)_p_/Al‐0.9Cu composite achieved an outstanding tensile strength at elevated temperatures up to 350°C. These results of the present study can set the stage for developing an efficient design of high‐performance Al alloys for high‐temperature structural applications.

## 2 Results and Discussion

**Figure 1:**
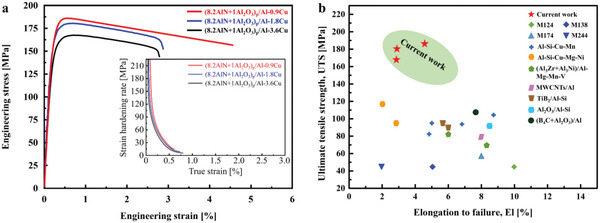
Mechanical properties of the (8.2AlN+1Al_2_O_3_)_p_/Al‐Cu composites at 350 °C. a) Engineering stress–strain curves for the (8.2AlN+1Al_2_O_3_)_p_/Al‐0.9Cu (red), (8.2AlN+1Al_2_O_3_)_p_/Al‐1.8Cu (blue), and (8.2AlN+1Al_2_O_3_)_p_/Al‐3.6Cu (black) composites. The inset shows the strain hardening rate *dσ*/*dε* (with *σ* and *ε* being the true stress and true strain, respectively) for the composites. b) Ultimate tensile strength plotted against elongation to failure at 350 °C for the (8.2AlN+1Al_2_O_3_)_p_/Al‐Cu composites and other Al alloys.^[^ ^20^, ^21^, ^22^, ^23^, ^24^, ^25^, ^26^, ^27^ ^].^

We studied the (8.2AlN+1Al_2_O_3_)_p_/Al‐Cu composites with different Cu contents: (8.2AlN+1Al_2_O_3_)_p_/Al‐0.9Cu, (8.2AlN+1Al_2_O_3_)_p_/Al‐1.8Cu and (8.2AlN+1Al_2_O_3_)_p_/Al‐3.6Cu. **Figure** **1**a shows the measured uniaxial stress–strain curves of these three (8.2AlN+1Al_2_O_3_)_p_/Al‐Cu composites at 350°C. The (8.2AlN+1Al_2_O_3_)_p_/Al‐0.9Cu composite exhibited an ultimate tensile strength (UTS) of 187 MPa (Table S1, Supporting Information), as revealed by the red curve in Figure 1a. Addition of Cu to 1.8 wt.% led to a slight decrease in the tensile strength, while the ductility was reduced significantly, decreasing from about 4.6% for the (8.2AlN+1Al_2_O_3_)_p_/Al‐0.9Cu composite to about 2.9% for the (8.2AlN+1Al_2_O_3_)_p_/Al‐1.8Cu composite. Surprisingly, the addition of Cu to 3.6 wt% resulted in a further decrease in the tensile strength, as revealed by the black curve in Figure 1a. Compared to the (8.2AlN+1Al_2_O_3_)_p_/Al‐1.8Cu and (8.2AlN+1Al_2_O_3_)_p_/Al‐3.6Cu composites, the (8.2AlN+1Al_2_O_3_)_p_/Al‐0.9Cu composite exhibited much stronger hardening rates under the same strain conditions, as shown in the inset in Figure 1a. A direct comparison of UTS of the current (8.2AlN+1Al_2_O_3_)_p_/Al‐Cu composites with those of other Al alloys is given in Figure 1b. The (8.2AlN+1Al_2_O_3_)_p_/Al‐Cu composites exhibited exceptional UTS that surpassed those of the mostly reported Al alloys. We note that the mechanical properties at 350 °C of the current (8.2AlN+1Al_2_O_3_)_p_/Al‐Cu composites were strongly dependent on the concentration of Cu. The current (8.2AlN+1Al_2_O_3_)_p_/Al‐Cu composites have better tensile strength than those of (8.2AlN+1Al_2_O_3_)_p_/Al composites, as reported in our previous work,^[^ ^19^ ^]^ and a higher Cu content (>0.9 wt.%) deteriorate the mechanical properties, causing a significant decrease in ductility.

**Figure 2:**
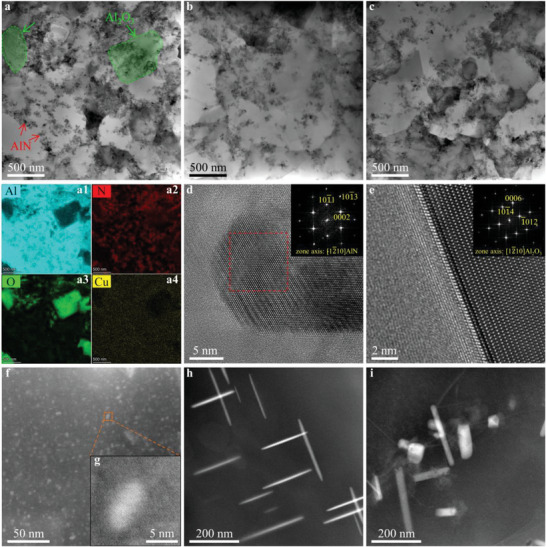
Microscopic structure. a) Bright‐field transmission electron microscopy (TEM) image of the (8.2AlN+1Al_2_O_3_)_p_/Al‐0.9Cu composite, and corresponding energy‐dispersive X‐ray spectroscopy (EDS) maps (a1, a2, a3, and a4) for individual elements Al, N, O, and Cu, showing uniformly distributed nano‐AlN (indicated by red arrows in a) and submicron‐Al_2_O_3_ (highlighted in light green, with its boundary indicated by the green dashed line in a) particles in the *α*‐Al matrix. b,c) Bright‐field TEM images of the (8.2AlN+1Al_2_O_3_)_p_/Al‐1.8Cu composite and the (8.2AlN+1Al_2_O_3_)_p_/Al‐3.6Cu composite, respectively, showing the uniform distribution and dispersion of nano‐AlN particles at the grain boundaries and within the *α*‐Al grains. d) High‐resolution TEM (HRTEM) image showing a characteristic interface between a nano‐AlN particle and the *α*‐Al matrix. The inset shows the selected area electron diffraction (SAED) pattern of the red dashed rectangle region in the main image, showing that the AlN particle had an *hcp* structure with a zone axis of [1$\bar{2}$10]. e) HRTEM image showing a characteristic interface between a submicron‐Al_2_O_3_ particle and the *α*‐Al matrix. The inset shows the corresponding SAED pattern of the main image, showing that the Al_2_O_3_ particle had an *hcp* structure with a zone axis of [1$\bar{2}$10]. f) High‐angle annular dark field (HAADF) image of the (8.2AlN+1Al_2_O_3_)_p_/Al‐0.9Cu composite (T6‐treated), showing the formation of a large quantity of Guinier–Preston (GP) zones in the *α*‐Al matrix. g) is the zoomed‐in image of orange dashed rectangle region in f), showing the nanocluster has a coherent lattice structure with the matrix. h, i) HAADF images showing the morphology and distribution of *θ'* (Al_2_Cu) precipitates in the (8.2AlN+1Al_2_O_3_)_p_/Al‐1.8Cu and (8.2AlN+1Al_2_O_3_)_p_/Al‐3.6Cu composites after T6 treatment, respectively.

To reveal the underlying mechanism of the strengthening effect, we studied the microstructures of the (8.2AlN+1Al_2_O_3_)_p_/Al‐Cu composites in detail using TEM. **Figure** **2**a shows the bright‐field TEM image and corresponding energy‐dispersive X‐ray spectroscopy (EDS) maps (a1–a4) of the (8.2AlN+1Al_2_O_3_)_p_/Al‐Cu composites. As shown in Figure 2a, nano‐AlN particles (indicated by red arrows) and submicron‐Al_2_O_3_ particles (highlighted in light green) were uniformly distributed and dispersed in the (8.2AlN+1Al_2_O_3_)_p_/Al‐0.9Cu matrix. This uniform dispersion of particles in the *α*‐Al matrix was also confirmed by TEM analysis of the (8.2AlN+1Al_2_O_3_)_p_/Al‐1.8Cu and (8.2AlN+1Al_2_O_3_)_p_/Al‐3.6Cu composites, as shown in Figure 2b,c, respectively, which clearly revealed that nano‐AlN particles were located in the *α*‐Al grain interiors and grain boundaries (GBs). The TEM microstructural characterization results shown in Figure 2a–c indicated that the average grain size of the *α*‐Al matrix was 0.74 ± 0.02 µm (Figure S1a–c,a1–c1, Supporting Information). Moreover, as shown in Figure 2d, the interfaces between the nano‐AlN particles and the *α*‐Al matrix were characterized at the atomic scale by HRTEM, and the inset shows the selected area electron diffraction (SAED) pattern of the red dashed rectangle region, which revealed that the AlN particle had an *hcp* structure probed from $\left[1\bar{2}10\right]$ zone axis. In addition, Figure 2e shows the interfaces between submicron‐Al_2_O_3_ particles and the *α*‐Al matrix, and the inset is the corresponding SAED pattern that shows the Al_2_O_3_ particle oriented to the $\left[1\bar{2}10\right]$ zone axis with an *hcp* structure. Figure S2a–c (Supporting Information) shows the scanning electron microscopy (SEM) images of the (8.2AlN+1Al_2_O_3_)_p_/Al‐Cu composites. The results shown in Figure S2d,e (Supporting Information) revealed the nano‐AlN and submicron‐Al_2_O_3_ particles had an average size of ≈50 nm and ≈457 nm, respectively.

Furthermore, the precipitate microstructures in the *α*‐Al matrix of the T6‐treated (8.2AlN+1Al_2_O_3_)_p_/Al‐Cu composites were characterized using high‐angle annular dark field (HAADF) imaging. It revealed that the (8.2AlN+1Al_2_O_3_)_p_/Al‐0.9Cu composite revealed an absence of *θ'* (Al_2_Cu) precipitates in the *α*‐Al matrix. Instead, a large density of nanoclusters (GP zones) formed with a relatively uniform and homogeneous distribution, as shown in Figure 2f, and their identity was confirmed by high‐magnification HAADF analysis (Figure 2g). However, for the (8.2AlN+1Al_2_O_3_)_p_/Al‐1.8Cu and (8.2AlN+1Al_2_O_3_)_p_/Al‐3.6Cu composites, the generated precipitate microstructures were very different from those produced in the (8.2AlN+1Al_2_O_3_)_p_/Al‐Cu matrix, where coarser and sparser *θ'* (Al_2_Cu) precipitates were present in the *α*‐Al matrix, as shown in Figure 2h,i.

**Figure 3:**
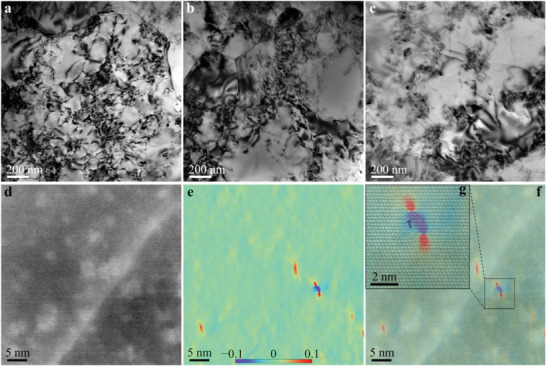
Plastic deformation mechanism. a–c) TEM images showing the dislocation microstructures in the samples of (8.2AlN+1Al_2_O_3_)_p_/Al‐0.9Cu, (8.2AlN+1Al_2_O_3_)_p_/Al‐1.8Cu, and (8.2AlN+1Al_2_O_3_)_p_/Al‐3.6Cu composites after plastic deformation at 350 °C, respectively, with the accumulation of large numbers of dislocations resulting from interactions with the nano‐AlN particles. d) HAADF image of the T6‐treated (8.2AlN+1Al_2_O_3_)_p_/Al‐0.9Cu composite after plastic deformation at 350 °C, showing the microstructure near the GP zones. e) Strain map of the HAADF image in (d), showing the relationship between the strain map and the distribution of GP zones after plastic deformation at 350 °C. f) Superimposition of the HAADF image (d) and strain map (e). g) Zoomed‐in image of the black dashed rectangle region in (f). A dislocation was present near the GP zone, as marked by the symbol “⊥”.

To investigate the deformation mechanisms, the microstructures of the specimens after plastic deformation at 350 °C were analyzed by TEM (**Figure** **3**). As shown in Figure 3a–c, large quantities of dislocations were curved and tangled in the matrix, suggesting that the nano‐AlN particles strongly suppressed dislocation propagation during plastic deformation. Furthermore, dislocation interactions and multiplication were complex, resulting in a high dislocation density. Moreover, the nano‐AlN particles located at the GBs contributed to strengthening of the composites by providing strong resistance to the GB sliding at elevated temperatures, which can be further demonstrated by the electron backscatter diffraction (EBSD) statistics on the change of the *α*‐Al grain size before and after tension at 350 °C (Figure S1, Supporting Information). A key question that needs to be answered is why the (8.2AlN+1Al_2_O_3_)_p_/Al‐0.9Cu composite achieved a combination of high tensile strength and ductility at 350 °C. As mentioned above, the main difference between the composites was the precipitates in the matrix (Figure 2f–i). To explicitly analyze the relationship between the GP zones and the plastic deformation, we analyzed the evolution of GP zones in the matrix of the (8.2AlN+1Al_2_O_3_)_p_/Al‐0.9Cu composite (T6‐treated) after plastic deformation at 350 °C, as shown in Figure 3d, and the corresponding GPA strain maps (see Experimental Section) are shown in Figure 3e–g. The results are shown in Figure 3e with a color scale showing the strain values from −0.1 to 0.1, where the positive and negative strains are related to the tension and compression regions, respectively. Evidently, the strain fields are highly localized in the GP zones. The GP zones imposed strains on the surrounding matrix, thus retarding the motion of dislocations.^[^ ^28^, ^29^ ^]^ As shown in Figure 3g, dislocations were present around the GP zones, indicating that the dislocation was pinned by the GP zone, which gave rise to strong strain hardening of the matrix (inset of the Figure 1a). Meanwhile, the low elastic misfit strain around the nanoscale GP zones, due to the small size of the GP zones and their coherent interfaces with the matrix, alleviated stress concentrations that would otherwise occur as the case for the coarse *θ'* precipitates. This effectively suppressed the nucleation of microcracks during deformation, which contributed to the large elongation. Therefore, the (8.2AlN+1Al_2_O_3_)_p_/Al‐0.9Cu composite had a concurrent high strength and high ductility at 350 °C.

## 3 Conclusion

In summary, our heat‐resistant Al–Cu composites, fabricated using a liquid–solid reaction method, contain submicron‐scale matrix grains with a uniform distribution of particles including nano‐AlN and submicron‐Al_2_O_3_. The UTS of the (8.2AlN+1Al_2_O_3_)_p_/Al‐0.9Cu composite at 350 °C is 187 MPa, which is much higher than those of the most known Al alloys. The elevated‐temperature deformation mechanism is dominated by nano‐AlN particles suppressing dislocation motion and inhibiting GB sliding during deformation, in addition to the GP zones in the *α*‐Al matrix promoting strain hardening. The findings of our study can provide guidance for industrial applications of novel Al–Cu composites with superior mechanical behaviors at elevated‐temperature service conditions.

## 4 Experimental Section

### Fabrication of Materials

The raw materials used in this study included commercial purity Al powders (average size of ≈30 µm) with a purity of 99.7% (all compositions quoted in this work are in wt.% (weight percent) unless otherwise stated), hexagonal boron nitride powders (average size of ≈2 µm) with a purity of 98.5%, hexagonal alpha‐Al_2_O_3_ particles (average size of ≈0.45 µm) with a purity of 99.9%, and pure Cu powders (average size of ≈1 µm) with a purity of 99.9%. The powders were mixed and compacted into cylindrical billets with diameters of 90 mm and heights of 300 mm by a cold isostatic pressing (CIP) machine under a pressure of 280 MPa. Next, composites with different Cu mass fractions were fabricated by liquid–solid reactions in a vacuum furnace, and the temperature of the liquid–solid reactions was 800 °C. Then, the obtained ingots were extruded at 500 °C with an extrusion ratio of 16:1. Subsequently, the extruded rods underwent T6 treatment (solution treatment at 520 °C for 2 h followed by water quenching; aging treatment at 150 °C for 5 h followed by cooling to room temperature in air) and were used for microstructural characterization and mechanical testing.

### Microstructural Characterization

High‐resolution transmission electron microscopy (HRTEM) by FEI Talos F200X at a voltage of 200 kV was used to characterize the microstructures and morphologies of the composites. The transmission electron microscopy (TEM) samples were first ground to 60 µm thickness by SiC abrasive papers, and punched into 3 mm diameter discs. Then, the discs were polished by ion beams using a Gatan 695 precision ion polishing system (PIPS) at 5 kV for 5 min. Strain mapping analysis was performed using the geometrical phase analysis (GPA) software installed in Digital Micrograph (Gatan).^[^ ^30^ ^]^

### Mechanical Testing

The T6‐treated Al composites along the hot extrusion direction were used for tension tests. The diameter and length of the tensile specimens were 10 and 50 mm, respectively (Figure S3, Supporting Information). Uniaxial tensile tests were carried out on a WDW–100D testing machine at a loading rate of 2 mm min^−1^. The specimens were heated up to 350 °C by using a resistive electromechanical‐system‐based heater, and the temperature was held for at least 30 min before starting the tensile test. Each composition of the specimen was tested repeatedly at least three times to ensure measurement accuracy.

## Conflict of Interest

The authors declare no conflict of interest.

## Supporting information

Supporting Information

## Data Availability

The data that support the findings of this study are available from the corresponding author upon reasonable request.

## References

[1] W. W. Sun , Y. M. Zhu , R. Marceau , L. Y. Wang , Q. Zhang , X. Gao , C. Hutchinson , Science 2019, 363, 972. 30819960 10.1126/science.aav7086

[2] C. S. Tiwary , P. Pandey , S. Sarkar , R. Das , S. Samal , K. Biswas , K. Chattopadhyay , Prog. Mater. Sci. 2022, 123, 100793.

[3] J. C. Williams , E. A. Starke , Acta Mater. 2003, 51, 5775.

[4] P. Xie , S. Y. Chen , K. H. Chen , H. B. Jiao , L. P. Huang , Z. Zhang , Z. Yang , Corros. Sci. 2019, 161, 108184.

[5] J. H. Zhao , Y. L. Deng , J. G. Tang , J. Zhang , J. Alloy. Compd. 2019, 809, 151788.

[6] M. Chemingui , K. Kassis , M. Khitouni , J. Masmoudi , A. W. Kolsi , IOP Conf. Ser.: Mater. Sci. Eng. 2010, 13, 012010.

[7] K. E. Knipling , D. N. Seidman , D. C. Dunand , Acta Mater. 2011, 59, 943.

[8] S. Roy , L. F. Allard , A. Rodriguez , W. D. Porter , A. Shyam , Metall. Mater. Trans. A 2017, 48, 2543.

[9] Y. Liu , R. A. Michi , D. C. Dunand , Mater. Sci. Eng. A 2019, 767, 138440.

[10] L. J. Zuo , B. Ye , J. Feng , H. X. Zhang , X. Y. Kong , H. Y. Jiang , Mater. Sci. Eng. A 2020, 772, 138794.

[11] M. Mantina , S. L. Shang , Y. Wang , L. Q. Chen , Z. K. Liu , Phys. Rev. B 2009, 80, 184111.

[12] M. E. van Dalen , R. A. Karnesky , J. R. Cabotaje , D. C. Dunand , D. N. Seidman , Acta Mater. 2009, 57, 4081.

[13] A. V. Krainikov , O. D. Neikov , Powder Metall. Met. Ceram. 2013, 51, 554.

[14] Z. Wang , R. T. Qu , S. Scudino , B. A. Sun , K. G. Prashanth , D. V. Louzguine‐Luzgin , M. W. Chen , Z. F. Zhang , J. Eckert , NPG Asia Mater. 2015, 7, e229.

[15] S. Kumar , S. K. Singh , J. Kumar , Q. Murtaza , Mater. Today: Proc 2018, 5, 3237.

[16] T. C. Lin , C. Z. Cao , M. Sokoluk , L. Jiang , X. Wang , J. M. Schoenung , E. J. Lavernia , X. C. Li , Nat. Commun. 2019, 10, 4124. 31511518 10.1038/s41467-019-12047-2 PMC6739343

[17] P. Kürnsteiner , P. Bajaj , A. Gupta , M. B. Wilms , A. Weisheit , X. S. Li , C. Leinenbach , B. Gault , E. A. Jägle , D. Raabe , Addit. Manuf. 2020, 32, 100910.

[18] C. Suwanpreecha , J. P. Toinin , R. A. Michi , P. Pandee , D. C. Dunand , C. Limmaneevichitr , Acta Mater. 2019, 164, 334.

[19] K. W. Xie , J. F. Nie , X. Ma , X. F. Liu , Mater. Charact. 2020, 170, 110672.

[20] MAHLE GmbH , Pistons and Engine Testing, 2nd ed., Springer, Fachmedien, Wiesbaden, Germany 2016.

[21] L. W. Pan , S. N. Zhang , Y. Yang , N. Gupta , C. Yang , Y. J. Zhao , Z. L. Hu , Metall. Mater. Trans. A 2020, 51, 214.

[22] Q. Liu , L. M. Ke , F. C. Liu , C. P. Huang , J. Mater. Eng. 2016, 44, 20.

[23] Y. N. Zan , Y. T. Zhou , Z. Y. Liu , Q. Z. Wang , W. G. Wang , D. Wang , B. L. Xiao , Z. Y. Ma , Mater. Sci. Eng. A 2020, 773, 138840.

[24] G. J. Li , H. C. Liao , X. J. Suo , Y. Y. Tang , U. S. Dixit , P. Petrov , Mater. Sci. Eng. A 2018, 709, 90.

[25] J. Feng , B. Ye , L. J. Zuo , R. J. Qi , Q. D. Wang , H. Y. Jiang , R. Huang , W. J. Ding , Mater. Sci. Eng. A 2017, 706, 27.

[26] G. Han , W. Z. Zhang , G. H. Zhang , Z. J. Feng , Y. J. Wang , Mater. Sci. Eng. A 2015, 633, 161.

[27] J. K. Xu , G. Chen , Z. Y. Zhang , Y. T. Zhao , X. Zhou , Z. L. Zhang , M. G. Meng , X. Liu , Q. Yan , Mater. Res. Express 2018, 5, 116502.

[28] Y. Chen , M. Weyland , C. R. Hutchinson , Acta Mater. 2013, 61, 5877.

[29] C. R. Hutchinson , F. de Geuser , Y. Chen , A. Deschamps , Acta Mater. 2014, 74, 96.

[30] M. J. Hÿtch , E. Snoeck , R. Kilaas , Ultramicroscopy 1998, 74, 131.

