# Supplementary material for: A Novel Al–Cu Composite with Ultra‐High Strength at 350 °C via Dual‐Phase Particle Reinforced Submicron‐Structure
Source: Adv Sci (Weinh). 2023 Jul 11;10(25):2207208. doi: 10.1002/advs.202207208 (PMC10477875; doi:10.1002/advs.202207208)
Supplement: Supplementary file 1 — Supporting Information [file ADVS-10-2207208-s001.pdf]

ADVANCED SCIENCE
Open Access

Supporting Information

for *Adv. Sci.*, DOI 10.1002/advs.202207208

A Novel Al–Cu Composite with Ultra-High Strength at 350 °C via Dual-Phase Particle Reinforced Submicron-Structure

Kewei Xie, Jinfeng Nie, Chang Liu, Wenhao Cha, Ge Wu*, Xiangfa Liu* and Sida Liu**

Supporting Information

A Novel Al-Cu Composite with Ultra-High Strength at 350 °C via Dual-Phase Particle Reinforced Submicron-Structure

Kewei Xie, Jinfeng Nie, Chang Liu, Wenhao Cha, Ge Wu*, Xiangfa Liu*, Sida Liu**

Correspondence to: niejinfeng@njust.edu.cn (J.F. Nie), gewuxjtu@xjtu.edu.cn (G. Wu), xfliu@sdu.edu.cn (X.F. Liu), sidaliu@sdu.edu.cn (S.D. Liu)

This Word file includes:

Figure S1 to S6

Table S1 to S2

Supplementary Note 1 to 3

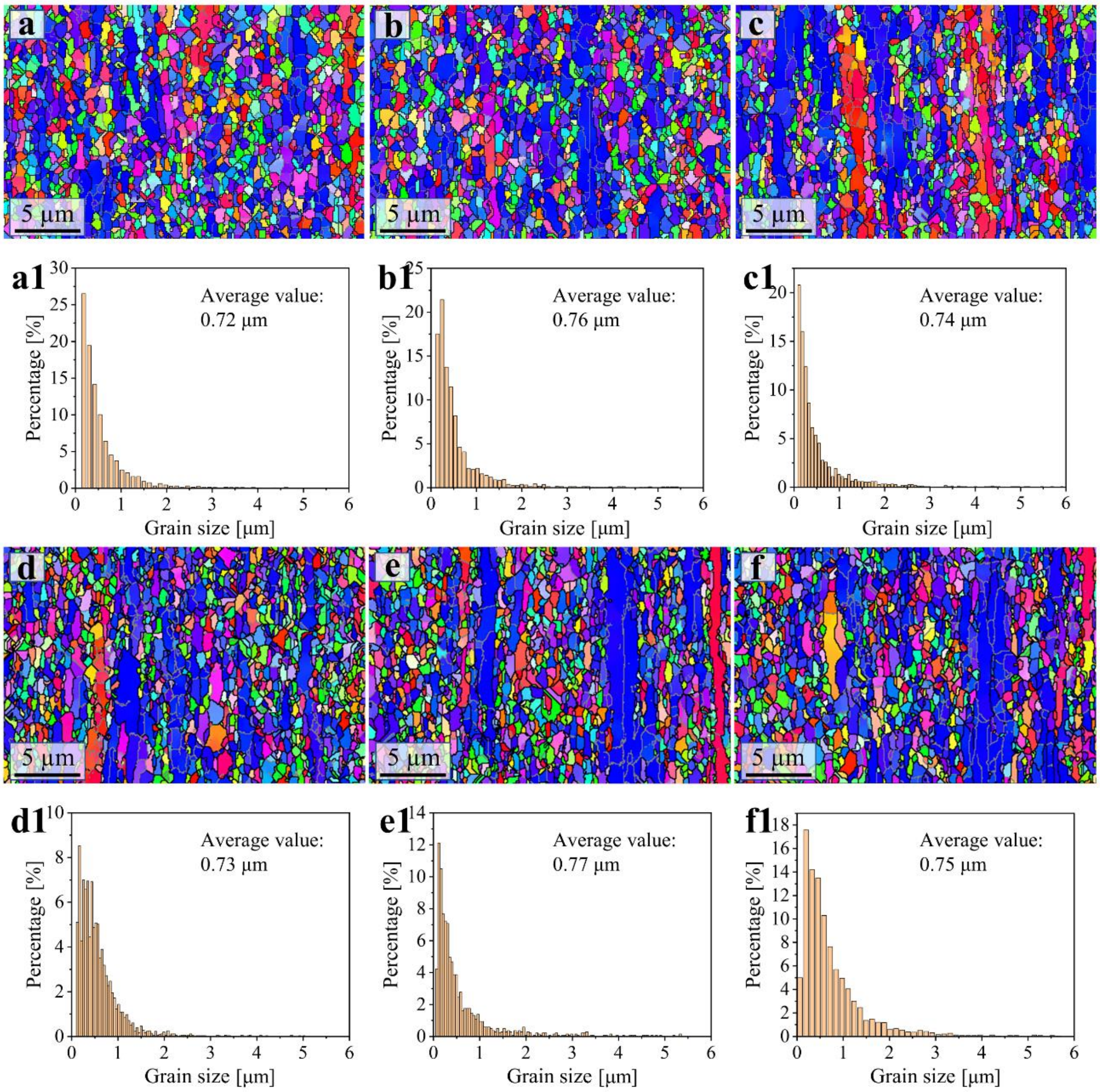

Figure S1. EBSD characterization of the α-Al grain size before (a–c and a1–c1) and after (d–f and d1–f1) tensile testing at 350 °C, (a, a1 and d, d1) $(8.2AlN+1Al_2O_3)_p$/Al-0.9Cu, (b, b1 and e, e1) $(8.2AlN+1Al_2O_3)_p$/Al-1.8Cu, and (c, c1 and f, f1) $(8.2AlN+1Al_2O_3)_p$/Al-3.6Cu. According to the average values of the α-Al grain size, no evident grain growth was observed in the α-Al matrix of the $(8.2AlN+1Al_2O_3)_p$/Al-Cu composites after plastic deformation at 350 °C, indicating that the nano-AlN particles effectively inhibited grain boundary sliding and had a strong pinning effect on the grain boundaries.

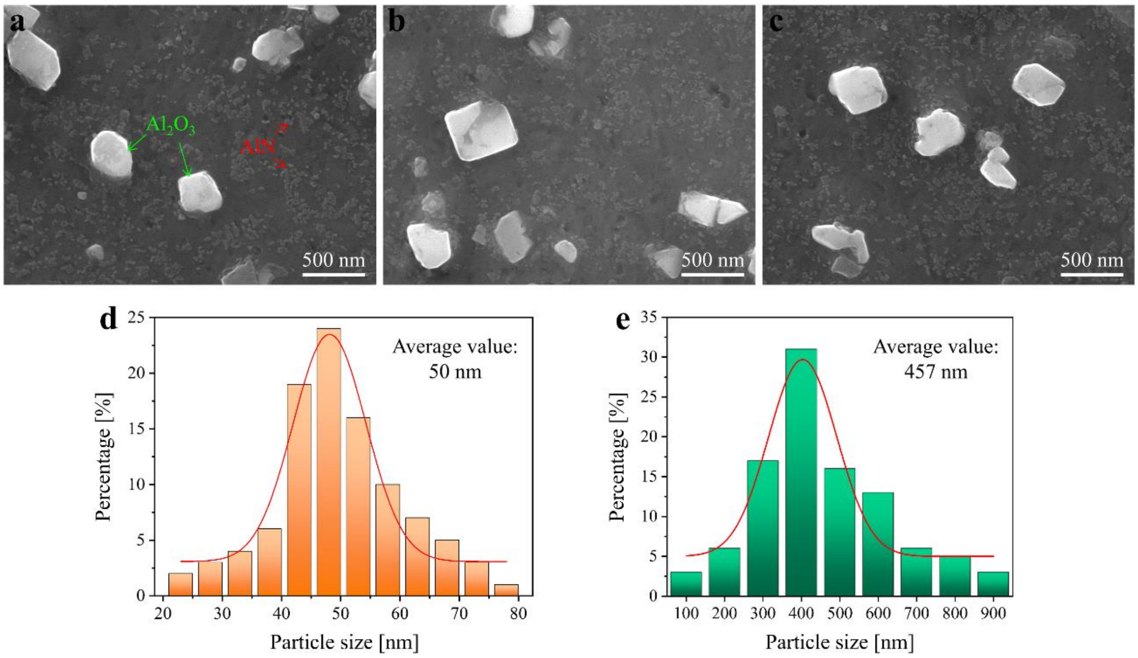

Figure S2. Microscopic structure. (a–c) SEM images of the $(8.2AlN+1Al_2O_3)_p/Al$-0.9Cu composite, $(8.2AlN+1Al_2O_3)_p/Al$-1.8Cu composite and the $(8.2AlN+1Al_2O_3)_p/Al$-3.6Cu composite, respectively, showing uniformly distributed AlN and Al_2O_3 particles in the α-Al matrix, which is consistent with the TEM characterization results. (d) A statistical histogram showing the size distribution of AlN particles. (e) A statistical histogram showing the size distribution of Al_2O_3 particle. The statistical analysis shows the uniform distribution of nano-AlN particles with an average size of 50 nm and submicron-Al_2O_3 particle with an average size of 457 nm, respectively.

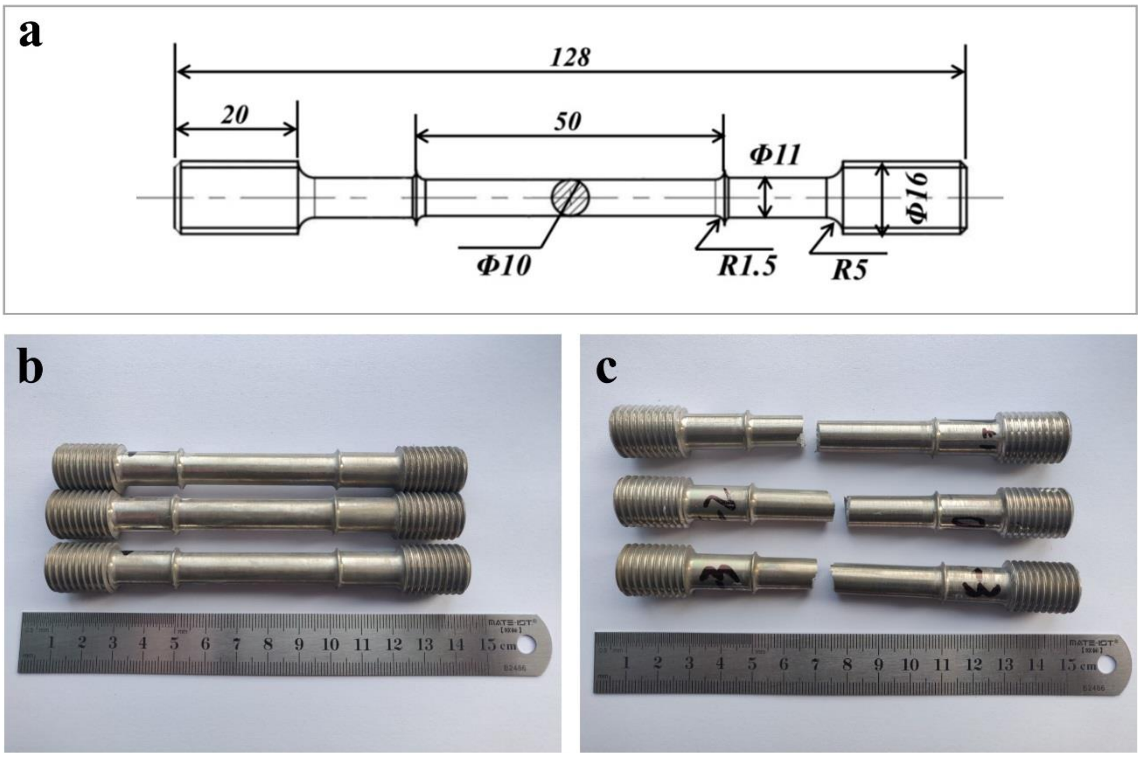

Figure S3. Tensile sample: (a) sample geometry for the tensile tests, with all dimensions in mm, and the specimens before (b) and after (c) tensile testing at 350 °C.

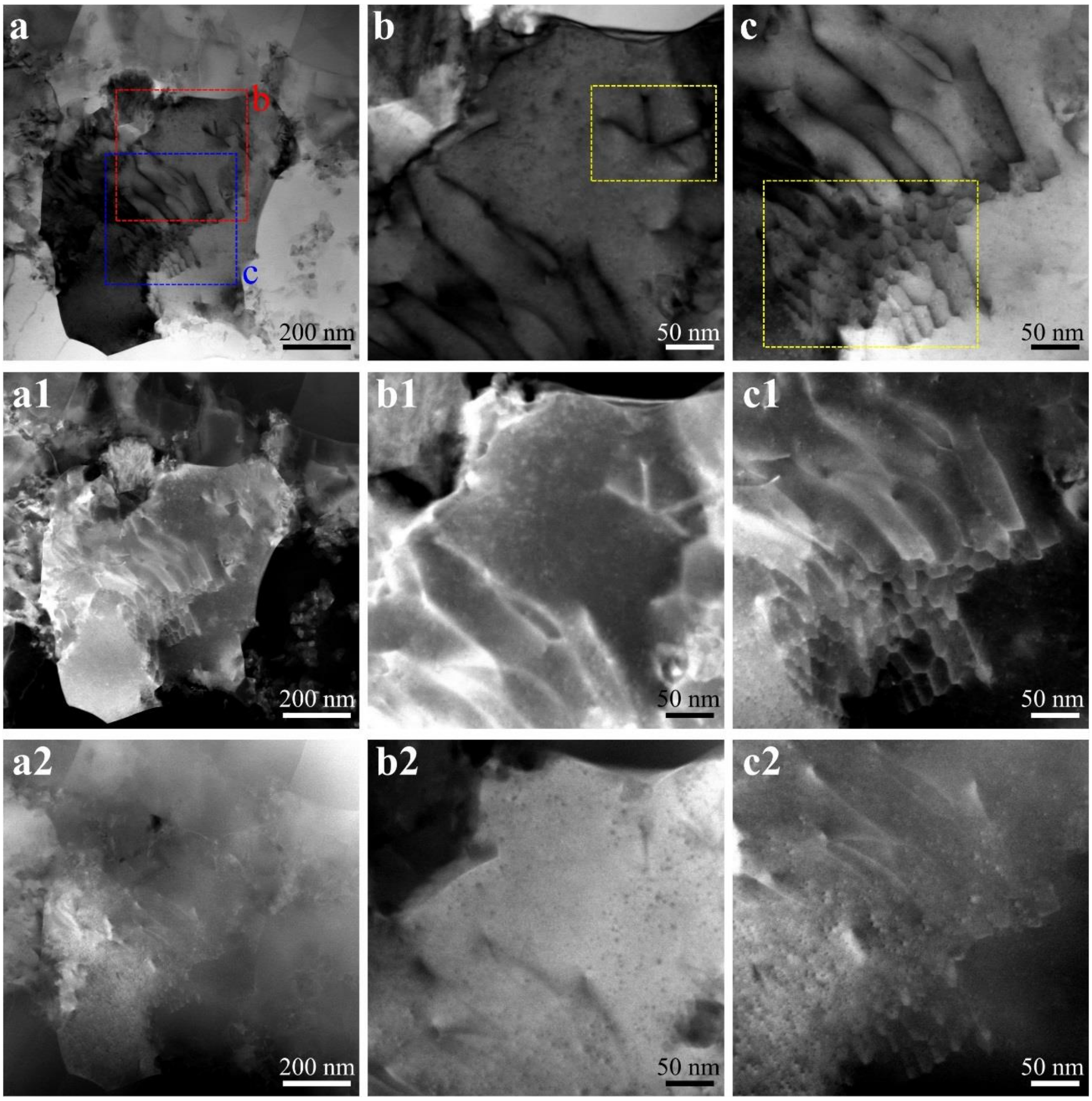

Figure S4. TEM images of the T6-treated $(8.2AlN+1Al_2O_3)_p$/Al-0.9Cu composite after plastic deformation at 350 °C, showing a high density of dislocations resulting from interactions with the GP zones existing in the α-Al matrix. (a), (b), and (c) are BF images; (b) and (c) are the zoomed-in images of the red and blue dashed rectangle regions in (a), respectively; (a1), (b1), and (c1) are HAADF images; (a2), (b2), and (c2) are DF images.

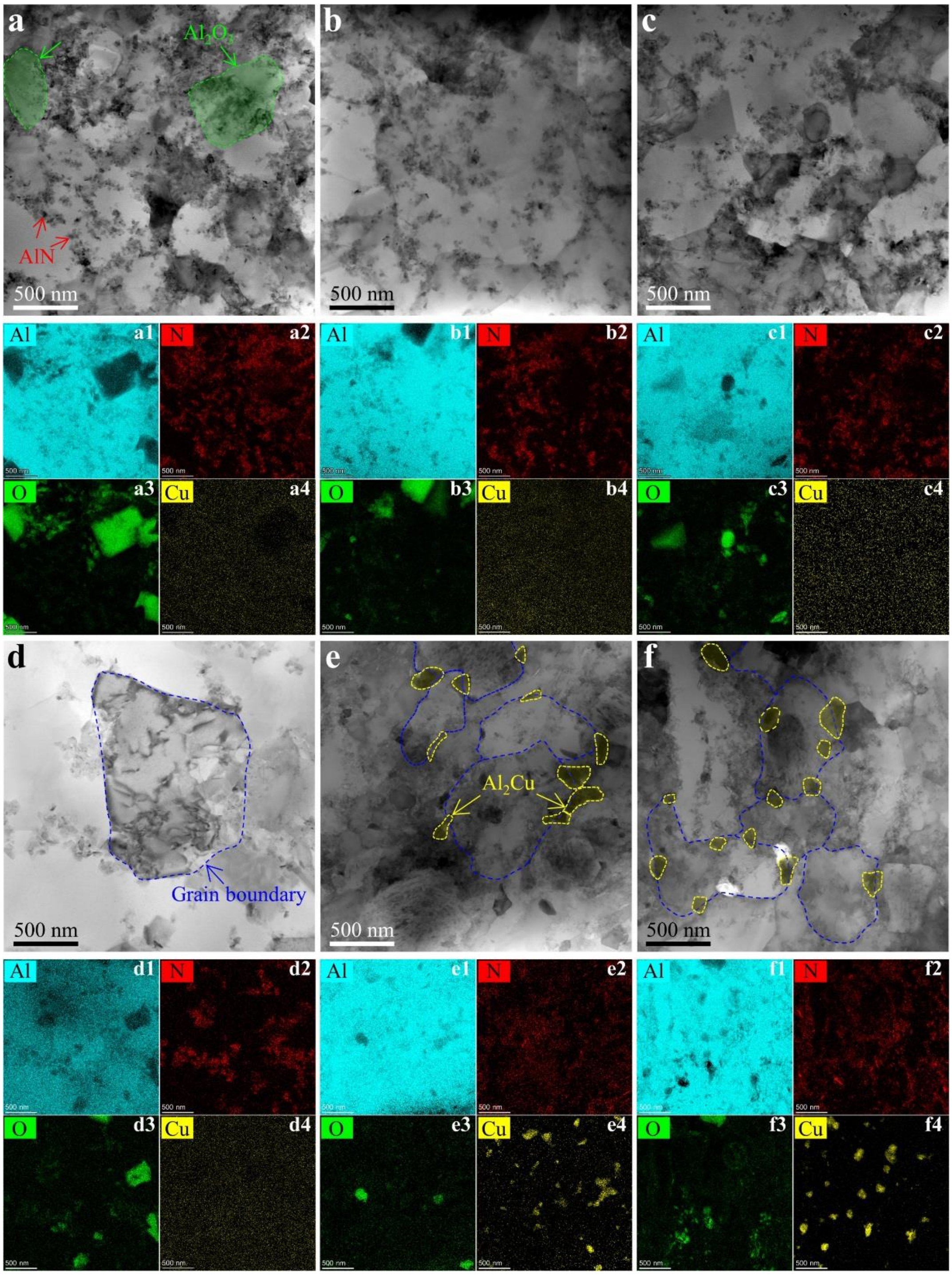

Figure S5. TEM images of the composites before (a, b, and c) and after (d, e, and f) plastic deformation at 350 °C, and their corresponding EDS maps for individual elements Al, N, O, and Cu, respectively. (a, d) $(8.2AlN+1Al_2O_3)_p$/Al-0.9Cu, (b, e) $(8.2AlN+1Al_2O_3)_p$/Al-1.8Cu, (c, f) $(8.2AlN+1Al_2O_3)_p$/Al-3.6Cu.

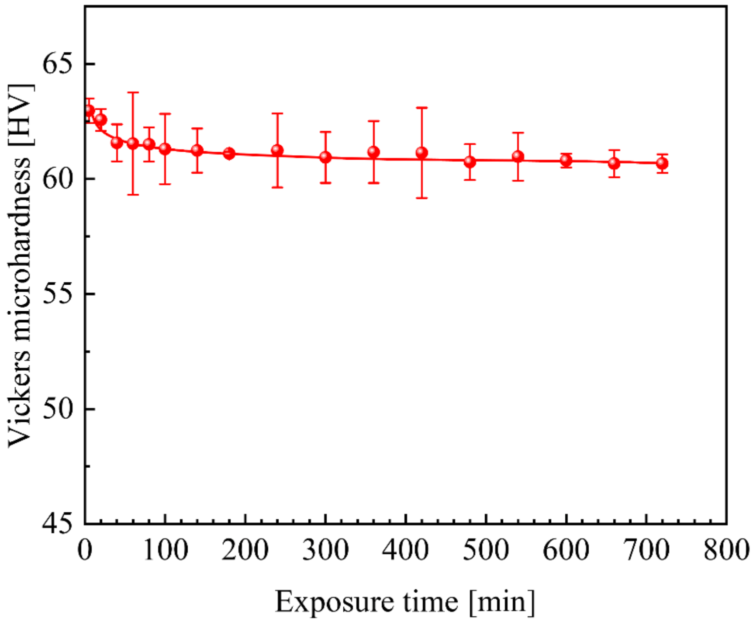

Figure S6. Vickers micro-hardness evolution with exposure time at 350 °C of the $(8.2AlN+1Al_2O_3)_p$/Al-0.9Cu composite. The error bars are the standard deviations of the mean.

Table S1. Mechanical properties of the prepared $(8.2AlN+1Al_2O_3)_p$/Al-Cu composites at 350 °C.

Composite	UTS [MPa]	YS [MPa]	EI [%]
$(8.2AlN+1Al_2O_3)_p$/Al-0.9Cu	187±3	184±2	4.6±0.2
$(8.2AlN+1Al_2O_3)_p$/Al-1.8Cu	180±2	176±3	2.9±0.3
$(8.2AlN+1Al_2O_3)_p$/Al-3.6Cu	168±4	162±3	2.8±0.2

Table S2. Vickers micro-hardness values of the $(8.2AlN+1Al_2O_3)_p$/Al-0.9Cu composite with exposure time at 350 °C.

Exposure time [min]	Vickers micro-hardness [HV]	Standard deviation
5	63.0	0.53
20	62.6	0.48
40	61.6	0.81
60	61.5	2.22
80	61.5	0.75
100	61.3	1.53
140	61.2	0.96
180	61.1	0.08
240	61.2	1.60
300	60.9	1.10
360	61.2	1.35
420	61.1	1.96
480	60.7	0.78
540	60.9	1.04
600	60.8	0.29
660	60.7	0.59
720	60.7	0.40

Supplementary Notes

Supplementary Note 1. Pinning effect of GP zones on dislocations

The result in Figure S4 shows a large volume fraction of GP zones in the Al matrix (as shown in Figure S4 b1, b2, c1, and c2), and a large number of dislocations are generated in the α-Al matrix (as shown in Figure S4b, b1, b2, c, c1, and c2). Some of the dislocations are straight and many of them are in curved and deflected morphologies, as shown in the top right of Figure S4b and the bottom of Figure S4c (highlighted by yellow dashed lines), indicating a pinning effect induced dislocation multiplication.

Supplementary Note 2. Coarse Al_2Cu particles distributed along grain boundaries are harmful to properties

Before starting the tensile test and during the holding period at 350 °C, the metastable θ' (Al_2Cu) precipitates in the matrix of composites with higher Cu content are more prone to be coarsened and transformed into large-sized equilibrium θ (Al_2Cu) particles. As shown in Figure S5e and f, these coarse Al_2Cu particles are distributed along grain boundaries, deteriorating the grain boundary strength under high temperatures. This effect leads to nucleation of voids and cracks along the grain boundaries, and thus reduces the strength and plasticity of the alloy. Based on the discussion above, the $(8.2AlN+1Al_2O_3)_p$/Al-1.8Cu and $(8.2AlN+1Al_2O_3)_p$/Al-3.6Cu composites show lower yield strength and UTS than that of the $(8.2AlN+1Al_2O_3)_p$/Al-0.9Cu composite at 350 °C.

Supplementary Note 3. Exposure time-dependent hardness at 350 °C

To further study the thermal resistance of the composites, we conducted experiments on the evolution of the $(8.2AlN+1Al_2O_3)_p$/Al-0.9Cu composite hardness with exposure time at 350 °C, using a high-temperature Vickers hardness test device (ZD-HVZ-10)

under the protection of high-purity (99.999%) argon gas. The specimen surfaces were metallographically polished with SiC sandpaper prior to indentation. The micro-hardness values were the average of at least three indents obtained by a Micro Vickers diamond indenter, with a load of 500 g and dwell time of 10 s. As indicated by the results in Figure S6 and Table S2, the hardness of the composite was ~ 63.0 HV after an exposure time of 5 min at 350 °C, and was then maintained stable even up to 720 min of exposure (corresponding to the hardness plateau of ~ 60.7 HV). Traditional heat-resistant aluminum alloys would soften significantly after heat exposure at 350 °C; however, the $(8.2AlN+1Al_2O_3)_p$/Al-0.9Cu composite did not show significant hardness degradation after heat exposure at 350 °C for up to 720 min. Therefore, based on the above, we are convinced that the $(8.2AlN+1Al_2O_3)_p$/Al-Cu composites have an excellently high thermal stability at 350 °C.
